# Safety of Intravitreal Injection of Stivant, a Biosimilar to Bevacizumab, in Rabbit Eyes

**DOI:** 10.18502/jovr.v15i3.7453

**Published:** 2020-07-29

**Authors:** Alireza Lashay, Hooshang Faghihi, Ahmad Mirshahi, Hassan Khojasteh, Alireza Khodabande, Hamid Riazi-Esfahani, Fahimeh Asadi Amoli, Elias Khalili Pour, Elham Delrish

**Affiliations:** ^1^Translational Ophthalmology Research Center, Farabi Eye Hospital, Tehran University of Medical Sciences, Tehran, Iran

**Keywords:** Biosimilar, Intravitreal Injection, Safety, Stivant

## Abstract

**Purpose:**

To evaluate the safety of intravitreal injection of Stivant, a biosimilar to bevacizumab, in rabbits using electrophysiological and histological analysis.

**Methods:**

Both eyes of 41 New Zealand albino rabbits were injected with 0.1 mL (2.5 mg) of Stivant. The rabbits were scheduled to be sacrificed 1, 2, 7, 14, and 28 days after injection for histopathological evaluations. Clinical examinations and electroretinography (ERG) were performed at baseline and just before sacrificing the rabbits. Fourteen separate rabbits received a reference drug (Avastin) and were considered as the control group. Furthermore, three other rabbits received the same volume of saline (saline control group). Rabbits of both control groups were sacrificed four weeks after injection. ERG was performed 1, 2, 7, 14, and 28 days after injections.

**Results:**

No significant difference was observed in a- and b-wave amplitudes and latency after intravitreal Stivant injection between baseline and different time points. Moreover, there was no statistically significant difference in wave amplitudes and latency between the Stivant and control groups. The histology of rabbit eyes of the Stivant and control groups after intravitreal injections was not distinguishable.

**Conclusion:**

The biosimilar Stivant, up to a dose of 2.5 mg, did not appear to be toxic to the retina in albino rabbits. These results suggest that this drug could be a safe and inexpensive alternative to intravitreal bevacizumab. The efficacy of these injections was not investigated in this study and needs to be evaluated in future studies.

##  INTRODUCTION

Vascular endothelial growth factor (VEGF) plays a substantial role in angiogenesis and vasculogenesis. Therefore, it is the main target for the treatment of cancers and ophthalmic vascular disorders.^[1,2]^ The inhibition of VEGF causes regression of aberrant new vessels in experimental models of proliferative vascular retinopathies and neovascularization of the choroid.^[3]^

In 2004, bevacizumab, a humanized full-length immunoglobulin G against VEGF, was approved by the US Federal Drug Administration for the management of colon cancer.^[4]^ Bevacizumab is now being used as an “off-label” intravitreal agent for wet age-related macular degeneration (AMD), diabetic retinopathy, and retinal vein occlusions globally.^[5]^ Intravitreal injections of ranibizumab and aflibercept are both approved for wet-type AMD and macular edema due to retinal vascular disorders. However, despite similar efficacy to bevacizumab, there are large differences between the costs of these drugs. On an average, bevacizumab is 20 times less expensive than ranibizumab and aflibercept.^[6]^

A biosimilar is “essentially the same” as a reference biologic in terms of structure, efficacy, safety, and quality, although it has some natural variability owing to its complex nature and production methods.^[7]^ Biosimilars, which are economically more viable, have the potential to reduce healthcare costs relative to reference biologics, thereby increasing treatment access for patients who need them.^[8,9]^ Stivant (CinnaGen Co., Tehran, Iran) has been developed as a biosimilar to a reference product, bevacizumab (Avastin: Genentech, Inc., South San Francisco, CA). Stivant is formulated with the same excipients as the reference product and is provided in the same pharmaceutical form and dosage strength. AvastinⓇ and Stivant have similar safety and efficacy as intravenous injections for metastatic colorectal cancer and have been approved for this use.

To determine the safety of intravitreal injection of this biosimilar drug, the albino rabbits were injected intravitreally with Stivant and evaluated for any functional and histological changes in the retina.

##  METHODS

Forty-one albino New Zealand rabbits, weighing between 1.5 and 2.5 kg, were used to evaluate the safety of intravitreal Stivant injection. The rabbits were treated in agreement with the statement for the use of animals in ophthalmic and vision research, proposed by The Association for Research in Vision and Ophthalmology. The study design was approved by the Institutional Review Board of Tehran University of Medical Sciences.

The rabbits were kept in an air-conditioned room with a 12-hour light–dark cycle and fed with standard processed laboratory food. Seven and eight of the rabbits were scheduled to be sacrificed 24 and 48 hours after injection, respectively, and the remaining were divided into three separate groups to be sacrificed at one (seven rabbits), two (ten rabbits), and four weeks (nine rabbits) after injection. Clinical examinations and electroretinography (ERG) were performed at baseline and just before killing the rabbits (Table 1). Fourteen separate rabbits received a reference drug (Avastin: Genentech, Inc., South San Francisco, CA) and were considered as the control group. Furthermore, three other rabbits received the same volume of normal saline and were considered as the saline control group. Both control groups were sacrificed four weeks after injection; however the ERG for these groups was performed 1, 2, 7, 14, and 28 days after injections (Table 1).

**Table 1:** Number of rabbits in each group


	**Number of rabbits**	**Time for enucleation and scarification**
**Group 1 (Stivant)**	7	1 day after injection
**Group 2 (Stivant)**	8	2 days after injection
**Group 3 (Stivant)**	7	1 week after injection
**Group 4 (Stivant)**	10	2 weeks after injection
**Group 5 (Stivant)**	9	4 weeks after injection
**Control (Saline)**	3	4 weeks after injection
**Control (Avastin)**	14	4 weeks after injection
	
	

**Table 2:** The amplitudes and latency times of ERG waves from the Stivant-treated rabbits


**Groups**	**a-wave Amplitude**	**b-wave Amplitude**	**a-wave Latency Time**	**b-wave Latency Time**
**24 hours (14 eyes)** *				
**Pre-treatment**	–38.5 ± 5.5 µV **	97.1 ± 12 µV	13.9 ± 0.6 ms	32.6 ± 3.3 ms
**Post-treatment**	–36.1 ± 3.4 µV	99.1 ± 22.1 µV	13.9 ± 0.7 ms	32.3 ± 3.1 ms
**Difference in**	Decrease 6.2%	Increase 2.1%	Increase 0.1%	Decrease 1%
**48 hours (16 eyes) %**				
**Pre-treatment**	–34.8 ± 3.6 µV	91.7 ± 9.5 µV	13.5 ± 0.5 ms	33.6 ± 2.7 ms
**Post-treatment**	–32.6 ± 5.9 µV	98.8 ± 11.9 µV	14.1 ± 0.5 ms	35.1 ± 3.4 ms
**Difference in**	Decrease 6.4%	Increase 7.2%	Increase 4.3%	Increase 4.3%
**1 week (14 eyes) %**				
**Pre-treatment**	–47.2 ± 6 µV	128.5 ± 21.1 µV	13.9 ± 0.6 ms	35.3 ± 2.6 ms
**Post-treatment**	–37.9 ± 9.2 µV	127.2 ± 38.3 µV	14.8 ± 0.7 ms	37.3 ± 1.9 ms
**Difference in**	Decrease 19.8%	Decrease 1.1%	Increase 6.1%	Increase 5.4%
**2 weeks (20 eyes) %**				
**Pre-treatment**	–48.2 ± 10.3 µV	140.3 ± 21.7 µV	14.1 ± 0.7 ms	36.6 ± 0.9 ms
**Post-treatment**	–47.7 ± 9.5 µV	147.5 ± 21.5 µV	14.3 ± 0.6 ms	37.7 ± 1.1 ms
**Difference in**	Decrease 1.1%	Increase 4.9%	Increase 1.4%	Increase 3%
**4 weeks (18 eyes) %**				
**Pre-treatment**	–46.5 ± 7.1 µV	127.9 ±22.7 µV	14.7 ± 0.7 ms	36.1 ± 1.3 ms
**Post-treatment**	–48.6 ± 14.1 µV	115.3 ± 30.1 µV	15.1 ± 0.5 ms	37 ± 2.1 ms
**Difference in %**	Increase 4.4%	Decrease 9.9%	Increase 2.7%	Increase 2.5%
After injections; **Data are expressed as mean ± SD *Microvolts* (µV); Milliseconds (ms)				

**Table 3:** The amplitudes and latency times of ERG waves from the Avastin-treated rabbits (28 eyes)


**Groups**	**a-wave Amplitude**	**b-wave Amplitude**	**a-wave Latency Time**	**b-wave Latency Time**
**24 hours (** ***n*** = 28) *				
**Pre-treatment**	–37.8 ± 3 µV **	120.1 ± 14.7 µV	14.5 ± 0.5 ms	34.4 ± 1.5 ms
**Post-treatment**	–35.8 ± 2.8 µV	114.8 ± 13.8 µV	13.9 ± 0. 7 ms	35.1 ± 1.3 ms
**Difference in**	Decrease 5.3%	Decrease 4.5%	Decrease 4.2%	Increase 2%
**48 hours (** ***n*** ** = 28) %**				
**Pre-treatment**	–37.8 ± 3 µV	120.1 ± 14.7 µV	14.5 ± 0.5 ms	34.4 ± 1.5 ms
**Post-treatment**	–34.7 ± 5.9 µV	100.8 ± 13.6 µV	14.7 ± 0.3 ms	37.3 ± 1 ms
**Difference in**	Decrease 8.3%	Decrease 16.6%	Increase 1.3%	Increase 8.4%
**1 week (** ***n*** ** = 28) %**				
**Pre-treatment**	–37.8 ± 3 µV	120.1 ± 14.7 µV	14.5 ± 0.5 ms	34.4 ± 1.5 ms
**Post-treatment**	–32.9 ± 5.8 µV	106.5 ± 21.7 µV	14.3 ± 0.9 ms	32.5 ± 2.3 ms
**Difference in**	Decrease 13 %	Decrease 11.4%	Decrease 1.4%	Decrease 5.6%
**2 weeks (** ***n*** ** = 28) %**				
**Pre-treatment**	–37.8 ± 3 µV	120.1 ± 14.7 µV	14.5 ± 0.5 ms	34.4 ± 1.5 ms
**Post-treatment**	–44.5 ± 9.9 µV	134.4 ± 22.1 µV	13.6 ± 0.5 ms	35.1 ± 1.1 ms
**Difference in**	Increase 17%	Increase 11%	Decrease 6.3%	Increase 2%
**4 weeks (** ***n*** ** = 28) %**				
**Pre-treatment**	–37.8 ± 3 µV	120.1 ± 14.7 µV	14.5 ± 0.5 ms	34.4 ± 1.5 ms
**Post-treatment**	–42.6 ± 10.5 µV	110.4 ± 14.7 µV	14.2 ± 0.2 ms	33.2 ± 2.6 ms
**Difference in %**	Increase 12.6%	Decrease 8.1%	Decrease 2.1%	Decrease 3.5%
*After injections; **Data are exprssed as mean ± SD Microvolts (µV); Milliseconds (ms)			

**Table 4:** The amplitudes and latency times of ERG waves from the Saline-treated rabbits (6 eyes)


**Groups**	**a-wave Amplitude**	**b-wave Amplitude**	**a-wave Latency Time**	**b-wave Latency Time**
**24 hours (** ***n*** = 6) *				
**Pre-treatment**	–34.6 ± 2.1 µV **	114.8 ± 17.7 µV	12.4 ± 1.1 ms	33.2 ± 2.1 ms
**Post-treatment**	–32.9 ± 7.8 µV	106.4 ± 12.7 µV	13.2 ± 0.1 ms	34.2 ± 0.3 ms
**Difference in **	Decrease 5%	Decrease 6.6%	Increase 6.4%	Increase 3%
**48 hours (** ***n*** ** = 6) %**				
**Pre-treatment**	–34.6 ± 2.1 µV	114.8 ± 17.7 µV	12.4 ± 1.1 ms	33.2 ± 2.1 ms
**Post-treatment**	–33.5 ± 8.8 µV	101.4 ± 19.2 µV	14.7 ± 0.7 ms	35.6 ± 1.2 ms
**Difference in **	Decrease 3.2%	Decrease 11.7%	Increase 18.5%	Increase 7.2%
**1 week (** ***n*** ** = 6) %**				
**Pre-treatment**	–34.6 ± 2.1 µV	114.8 ± 17.7 µV	12.4 ± 1.1 ms	33.2 ± 2.1 ms
**Post-treatment**	–40 ± 8.1 µV	129.9 ± 27.2 µV	14.1 ± 0.6 ms	35.1 ± 1.6 ms
**Difference in **	Increase 15.6 %	Increase 13.1%	Increase 13.7%	Increase 5.7%
**2 weeks (** ***n*** ** = 6) %**				
**Pre-treatment**	–34.6 ± 2.1 µV	114.8 ± 17.7 µV	12.4 ± 1.1 ms	33.2 ± 2.1 ms
**Post-treatment**	–38.6 ± 8.4 µV	135.3 ± 28.3 µV	14.3 ± 0.7 ms	35.9 ± 1.5 ms
**Difference in **	Increase 11.5%	Increase 17.8%	Increase 15.3%	Increase 8.1%
**4 weeks (** ***n*** ** = 6) %**				
**Pre-treatment**	–34.6 ± 2.1 µV	114.8 ± 17.7 µV	12.4 ± 1.1 ms	33.2 ± 2.1 ms
**Post-treatment**	–41.5 ± 13.3 µV	110.3 ± 20.5 µV	14.1 ± 0.6 ms	35.2 ± 2.1 ms
**Difference in %**	Increase 19.8%	Decrease 4%	Increase 13.7%	Increase 6%
*After injections; **Data are expressed as mean ± SD Microvolts (µV); Milliseconds (ms)				

**Table 5:** Comparison of the amplitudes and latency times of rod responses from the Stivant-treated rabbits, saline-treated rabbits, and Avastin-treated rabbits


**Groups**		**a-wave Amplitude**	**b-wave Amplitude**	**a-wave Latency Time**	**b-wave Latency Time**
**24 hours**					
**Saline (** ***n*** * = 6)		–32.9 ± 7.8 µV	106.4 ± 12.7 µV	13.2 ± 0.1 ms	34.2 ± 0.3 ms
**Avastin (** ***n*** ** = 28)**		–35.8 ± 2.8 µV	114.8 ± 13.8 µV	13.9 ± 0. 7 ms	35.1 ± 1.3 ms
**Stivant (** ***n*** ** = 14)**		–36.1 ± 3.4 µV	99.1 ± 22.1 µV	13.9 ± 0.7 ms	32.3 ± 3.1 ms
**** ***P*** -value**	**Stivant/saline**	0.43	0.54	0.86	0.71
	**Stivant/Avastin**	0.73	0.21	0.79	0.69
**48 hours**					
**Saline (** ***n*** ** = 6)**		–33.5 ± 8.8 µV	101.4 ± 19.2 µV	14.7 ± 0.7 ms	35.6 ± 1.2 ms
**Avastin (** ***n*** ** = 28)**		–34.7 ± 5.9 µV	100.8 ± 13.6 µV	14.7 ± 0.3 ms	37.3 ± 1 ms
**Stivant (** ***n*** ** = 16)**		–32.6 ± 5.9 µV	98.8 ± 11.9 µV	14.1 ± 0.5 ms	35.1 ± 3.4 ms
**** ***P*** -value**	**Stivant/saline**	0.38	0.44	0.88	0.90
	**Stivant/Avastin**	0.33	0.29	0.81	0.76
**1 week**					
**Saline (** ***n*** ** = 6)**		–40 ± 8.1 µV	129.9 ± 27.2 µV	14.1 ± 0.6 ms	35.1 ± 1.6 ms
**Avastin (** ***n*** ** = 28)**		–32.9 ± 5.8 µV	106.5 ± 21.7 µV	14.3 ± 0.9 ms	32.5 ± 2.3 ms
**Stivant (** ***n*** ** = 14)**		–37.9 ± 9.2 µV	127.2 ± 38.3 µV	14.8 ± 0.7 ms	37.3 ± 1.9 ms
**** ***P*** -value**	**Stivant/saline**	0.19	0.33	0.87	0.53
	**Stivant/Avastin**	0.16	0.11	0.75	0.28
**2 weeks**					
**Saline (** ***n*** ** = 6)**		–38.6 ± 8.4 µV	135.3 ± 28.3 µV	14.3 ± 0.7 ms	35.9 ± 1.5 ms
**Avastin (** ***n*** ** = 28)**		–44.5 ± 9.9 µV	134.4 ± 22.1 µV	13.6 ± 0.5 ms	35.1 ± 1.1 ms
**Stivant (** ***n*** ** = 20)**		–47.7 ± 9.5 µV	147.5 ± 21.5 µV	14.3 ± 0.6 ms	37.7 ± 1.1 ms
**** ***P*** -value**	**Stivant/saline**	0.14	0.18	0.94	0.72
	**Stivant/Avastin**	0.81	0.09	0.61	0.66
**4 weeks**					
**Saline (** ***n*** ** = 6)**		–41.5 ± 13.3 µV	110.3 ± 20.5 µV	14.1 ± 0.6 ms	35.2 ± 2.1 ms
**Avastin (** ***n*** ** = 28)**		–42.6 ± 10.5 µV	110.4 ± 14.7 µV	14.2 ± 0.2 ms	33.2 ± 2.6 ms
**Stivant (** ***n*** ** = 18)**		–48.6 ± 14.1 µV	115.3 ± 30.1 µV	15.1 ± 0.5 ms	37 ± 2.1 ms
**** ***P*** -value**	**Stivant/saline**	0.14	0.68	0.88	0.67
	**Stivant/Avastin**	0.30	0.58	0.24	0.54
Data are expressed as mean ± SD *Number of the injected eyes in each group; **Based on generalized estimating equation (GEE) regression model *Microvolts* (µV); Milliseconds (ms)					

Rabbits with documented anterior or posterior segment abnormalities in the eye were excluded. The animals were anesthetized before all procedures using a mixture of xylazine hydrochloride (5 mg/kg) and ketamine hydrochloride (50 mg/kg). Before each examination, the pupil of the eyes was dilated by topical application of tropicamide 0.5% eye drop.

### Preparation and injection of the drugs

Under standard sterile conditions, standard tuberculin syringes with 29-gauge needles were filled with the drugs or saline. Intravitreal injections of the drugs in each group were performed after baseline examinations and baseline ERG. Rabbits were anesthetized with an intramuscular injection of the mixture of xylazine and ketamine. The eye was prepped with installation of the 5% diluted povidone iodine solution into the fornixes.

Both eyes of each rabbit in the Stivant and Avastin groups were injected with 0.1 mL (2.5 mg) of the drugs. Intravitreal injections into the mid-vitreous cavity were performed 1.5 mm posterior to the limbus. Anterior chamber paracentesis was done before intravitreal injections with a 29-gauge needle, withdrawing at least 0.05 mL of the aqueous fluid to prevent intraocular pressure rise after injections and also to prevent reflux from the injection site. The same volume (0.1 mL) of normal saline was injected into both eyes of each of the three control rabbits in the same manner. Ciprofloxacin and timolol eye drops were applied to the eyes for the first three days after injections.

### Clinical observations 

The eyes were examined clinically at baseline on the first and second days after injections and at the end of the first, second, and fourth weeks of injections just before enucleation. The following parameters were recorded: injection of the conjunctiva, status of the cornea, appearance of the crystalline lens, any pathologic findings in the retina, and any cell or flare in the anterior and posterior segments of the eye.

A hand-held slit lamp was used to evaluate the status of the anterior segment. The anterior chamber and vitreous cavity were carefully examined with the highest magnification to detect any cell or flare. At each follow-up, all eyes underwent indirect ophthalmoscopy.

### Electrophysiology

Electroretinography using the electrophysiological test system (Metrovision, France) was performed on both eyes of each rabbit at baseline and the time points previously mentioned. All rabbits underwent dark adaptation overnight before ERG tests and were prepared for the procedure under red light. The animals were anesthetized, and corneal surface anesthesia was achieved using tetracaine hydrochloride 0.5%. Flashlight intensity of 10 cdsm${}^{-2}$ was used for each recording; the average of the responses from four separate light stimuli was documented. ERG was recorded using a corneal contact lens electrode (Metrovision, France). The ground electrode was inserted into the ear, and the negative electrode was attached near the orbital rim.

The amplitude and implicit time measurements of the a- and b-waves were used to evaluate ERG responses. The a-wave amplitude was measured from the baseline to the first trough; the b-wave amplitude was measured from the a-wave trough to the peak of the b-wave. Latencies of the a- and b-waves were measured from the time of presenting the stimuli.

### Histopathologic examinations

After the last ophthalmoscopy and electrophysiology in each group, the animals were sacrificed by intravenous injection of 100 mg/kg of sodium pentobarbital while under deep anesthesia. With careful attention to prevent any damage to globe integrity, the eyes were enucleated. Then, each enucleated eye was promptly placed in a separate bottle containing the neutral formalin solution. After seven days, samples were fixed in paraffin. Microtome sections of 5-m thickness were prepared and stained with hematoxylin-eosin. Slides were examined under a light microscope.

### Statistical Analysis

Statistical analysis was performed using the SPSS software version 24.0. (IBM SPSS Statistics for Windows, Armonk, NY: IBM Corp). One-way analysis of variance was used to determine whether there were any statistically significant differences between the groups, based on the ERG parameters. The generalized estimating equation (GEE) regression model was used to analyze the effect of the injections on the ERG a- and b-wave amplitudes and implicit time between the Stivant and control groups, separately. A *P*-value < 0.05 was considered statistically significant.

##  RESULTS

Of 41 rabbits in the Stivant group, 3 were excluded due to death; 1 died just after injection, and the other 2 died five days and three weeks after injection, respectively. Stivant was tolerated well in the remaining rabbits. Two rabbits also died two weeks after injection in the Avastin control group. No obvious changes in food or water intake were observed.

### Clinical Evaluation 

At each anterior segment examination, there was no new significant abnormality in conjunctiva or different layers of the cornea. Anterior chamber or vitreous cells were not detected in any eye at different time points on biomicroscopic examination. There was no iris abnormality in any eye of each group. The crystalline lenses were clear. The vitreous, retina, choroid, and optic nerve seemed normal, based on indirect ophthalmoscopy.

### Electrophysiology

There was no significant difference between ERG wave amplitudes and implicit times between Stivant, Avastin, and saline groups at baseline. ERG changes were considered significant at each time point if the difference in amplitudes (a- and b-waves) was more than 20% of the baseline values.

The ERG results of the Stivant, Avastin, and saline groups are presented in Tables 2, 3, and 4, respectively. Dark-adapted bright flash ERG was performed for all rabbits before the intravitreal injection as a baseline standard and then at each time point. Despite a 19.8% decrease in the amplitude of the a-wave on day 7 after Stivant injection, no significant change was found in the a-wave amplitude and implicit time after injections in each group. We also observed a 13% reduction in the a-wave amplitude, one week after Avastin injection. These reductions were reversed just one week later in both groups.

This study did not show a significant change in the amplitude and latency of b-waves in Stivant and Avastin groups, although, when compared to the baseline, the b-wave amplitude decreased 9.9% and 8.1% in eyes that were evaluated four weeks after Stivant and Avastin injections, respectively.

There were no statistically significant differences between the Stivant, Avastin, and saline groups, based on ERG parameters. Also, based on the GEE regression model, there was no significant difference between the Stivant-injected eyes and the Avastin and saline control groups at each time point, separately. (Table 5)

### Histological Findings

Based on histopathological findings, there were no distinguishable changes in both the Stivant and control groups after intravitreal injections. There were no signs of ocular toxicity, based on histological evaluations in the groups.

In the light microscopic slides, there was no evidence of corneal deposits, thinning, or endothelial cell damage in the cornea. Uveal tissue did not show any inflammation or neovascularization. There was no sign of inflammation in the anterior and posterior segments of the eyes.

There was no evidence of intraocular hemorrhage in specimens, except the right eye of a rabbit that was sacrificed two weeks after Stivant injection. The right eye showed retinal and vitreous hemorrhage, although there was no significant change in ERG parameters from baseline in this eye.

All specimens had normal retinal thickness, ganglion cells, photoreceptor morphology, pigmented epithelial cells, and nuclear layers. No evidence of optic nerve edema, neuritis, or atrophy was identified.

**Figure 1:**
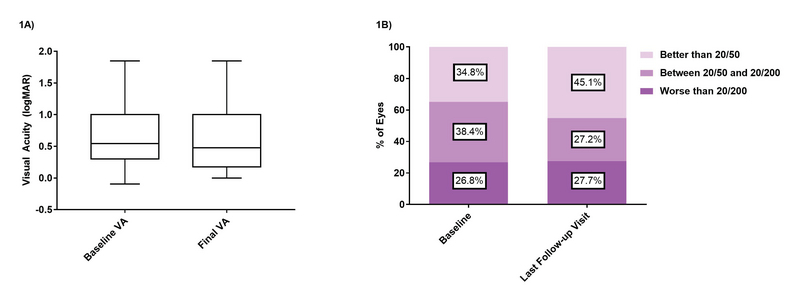
Representative histopathological sections from injected eyes four weeks after injection: there is preservation of the retinal cytoarchitecture without loss of the inner and outer nuclear layers or the inner and outer plexiform layers in both Avastin-injected (a) and Stivant-injected eyes (b) (Staining: hematoxylin and eosin [H&E], magnification: ×40).

The only positive histopathological finding was congestion of the choroid without hemorrhage in half of the eyes that were injected with Stivant, which were enucleated 24 hours after injection. This finding was not observed in Stivant-injected groups at other time points (Figure 1).

##  DISCUSSION

Our results showed that a single intravitreal injection of the biosimilar to bevacizumab (Stivant) at doses up to 2.5 mg in albino rabbit eyes did not result in apparent vitreoretinal toxicity at 1, 2, 7, 14, and 28 days after injection, based on electrophysiological and histopathological findings. The ERG responses of the experimental and two control group eyes were similar in a- or b-wave amplitudes and implicit times at different time points after injections.

Anti-VEGF agents play a key role in the management of different retinal conditions, such as wet AMD, diabetic macular edema, and retinal vein occlusion.^[10,11,12]^ As most of these patients need multiple anti-VEGF injections, these drugs incur high individual, medical, and societal costs.^[13]^ By decreasing the cost of therapy, the economic burden on the individual patients, their families, and society will be reduced. In developing countries, the high cost of treatment is an important limiting factor for patient compliance to anti-VEGF agents.^[13,14]^

Biosimilar drugs, in comparison with reference products, have the same structure, efficacy, safety, and quality, although there may be slight differences due to the complexities of the production process.^[7,8]^ As both the reference and biosimilar drugs have a degree of natural variability, studies are necessary to ensure that these differences do not affect the safety of intravitreal injection of these drugs. These biosimilar drugs can increase the community access to biological drugs, such as anti-VEGF agents, and can also reduce the burden on the healthcare budget.^[15]^

The biosimilar ranibizumab (RazumabⓇ; Intas Pharmaceuticals) is the first ophthalmic biosimilar that has been developed in India.^[7]^ It has undergone animal safety studies and larger humanized head-to-head studies to ensure close resemblance in pharmacokinetic and pharmacodynamics characteristics, safety, and efficacy to the reference drug. Intravitreal injection of Zybev (a biosimilar of bevacizumab) has also shown to be safe, based on different studies.^[16]^

The bevacizumab biosimilar (Stivant; CinnaGen Co) is the first biosimilar to an anti-VEGF agent that has been developed in Iran. Large studies in patients with systemic cancers have been conducted to ensure close resemblance in safety and efficacy with the reference product (AvastinⓇ). To assess the safety of intravitreal injection of this biosimilar drug, we performed intravitreal injections of Stivant at the double dosage in albino New Zealand rabbits and investigated the retinal function and histological findings. The intravitreal injection of bevacizumab may be potentially toxic to the eye through the following three mechanisms: first, the vehicle could be toxic itself; second, toxicity could be due to induction of an immune response by injecting an immunoglobulin; and third, toxicity could be due to interference with endogenous VEGF signaling.${}^{\left[27\right]}$

Manzano et al^[17]^ evaluated the safety of the intravitreal injection of the reference drug (AvastinⓇ). They defined at least a 30% reduction in ERG wave amplitudes to be significant, whereas we considered a lower threshold (20%) for ERG amplitude and latency changes to be significant. The ERG wave amplitude and latency changes were less than 10% at different time points in our study, except for a 19.8% reduction in a wave amplitude at week 1 after Stivant injection. This reduction was not observed two and four weeks after injections. One week after Avastin injection, we also observed a 13% reduction in the a-wave amplitude, which was reversed in the following weeks. These reductions may be due to a transient effect of the drugs on photoreceptors in the early post-injection period. We observed a similar but earlier reduction in the wave amplitude after saline injection; 48 hours post injection.

In this study, we report that intravitreal injection of a high dose of the biosimilar bevacizumab (Stivant) in rabbit eyes is well tolerated, at least in the short-term. The vitreous volume of a rabbit eye is approximately 1.5 mL and that of a human eye is approximately 5 mL. As the dose of 1.25 mg of bevacizumab is frequently used in humans, the doses of 1.25 and 2.5 mg of bevacizumab in rabbits result in approximately 3.3 and 6.6 times concentration of the medication in human eyes, respectively. Similar to some previous studies that evaluated the safety profile of a new anti-VEGF on the retinal tissue, we used 2.5 mg of this biosimilar drug to evaluate the safety of a higher concentration.^[9]^ However, the maximum safe dose of bevacizumab was not determined in this study.

This study has some limitations. The endpoint ERG of the study group did not have a significant change in parameters from baseline. However, this study evaluated the short-term changes after a single intravitreal injection and did not rule out the possibility that long-term follow-up, especially with more injections, might demonstrate inappropriate side effects. As ERG is primarily a functional test of the status of the photoreceptors and bipolar cells, normal ERG results do not exclude possible damage at the level of the retinal ganglion cells or their axons, although this was not seen in histological evaluations.^[18,19]^ Conversely, safety based on histological findings by light microscopy does not rule out possible changes at the submicroscopic level.^[20]^ Therefore, it is better to design a study to perform immunocytochemical analysis on the histopathologic sections to evaluate the possible damage to retinal microstructures. Another limitation of the study was the disparity between the study group and the control groups in terms of the number of anesthesia sessions before enucleation and lack of tissue for histopathological evaluation in the control groups at time points earlier than four weeks after injections.

In summary, a single intravitreal injection of the biosimilar to bevacizumab (Stivant) up to a dose of 2.5 mg (high concentration) in the eyes of albino rabbits did not appear to be toxic for the retina. These results suggest that this drug could be a safe and cost-effective alternative to the reference drug. However, further investigations are needed to evaluate the long-term safety and efficacy of this biosimilar drug.

##  Financial Support and Sponsorship

The study received support from the Eye Research Center, Farabi Eye Hospital, Tehran University of Medical Sciences, TUMS#38687.

##  Conflicts of Interest

There are no conflicts of interest.
